# Supplementary material for: A Combination of the Natural Molecules Gallic Acid and Carvacrol Eradicates P. aeruginosa and S. aureus Mature Biofilms
Source: Int J Mol Sci. 2022 Jun 27;23(13):7118. doi: 10.3390/ijms23137118 (PMC9266711; doi:10.3390/ijms23137118)
Supplement: Supplementary file 1 [file ijms-23-07118-s001.zip › ijms-1768110-supplementary.pdf]

Supplementary material for on-line submission

Supplementary Table S1: Maximal solubility of active ingredients in 75% EtOH or water.

	Active ingredients	G	K	Q
**Solution**	Solvent	H_2O	EtOH (75%)	EtOH (75%)
	Solubility limit (mg/mL)	10.0[a]	50.0[b]	5.0[c]

[a]After 5min at 50°C under agitation, [b]after manual mix, [c]after 20min at 50°C under agitation. ND – Not detected.

Supplementary Table S2: Maximal solubility of active ingredient combinations in 75% EtOH.

**Active ingredients combinations**	**GK**	**KQ**	**GQ**	**GKQ**
Solvent	EtOH (75%)			
Solubility limit (mg/mL)	10.0 – 50.0[a]	50.0 – 5.0[b]	10.0 – 5.0[b]	10.0 – 50.0 – 5.0[b]

[a]*After manual mix,* [b]*after 20min at 50°C under agitation.*

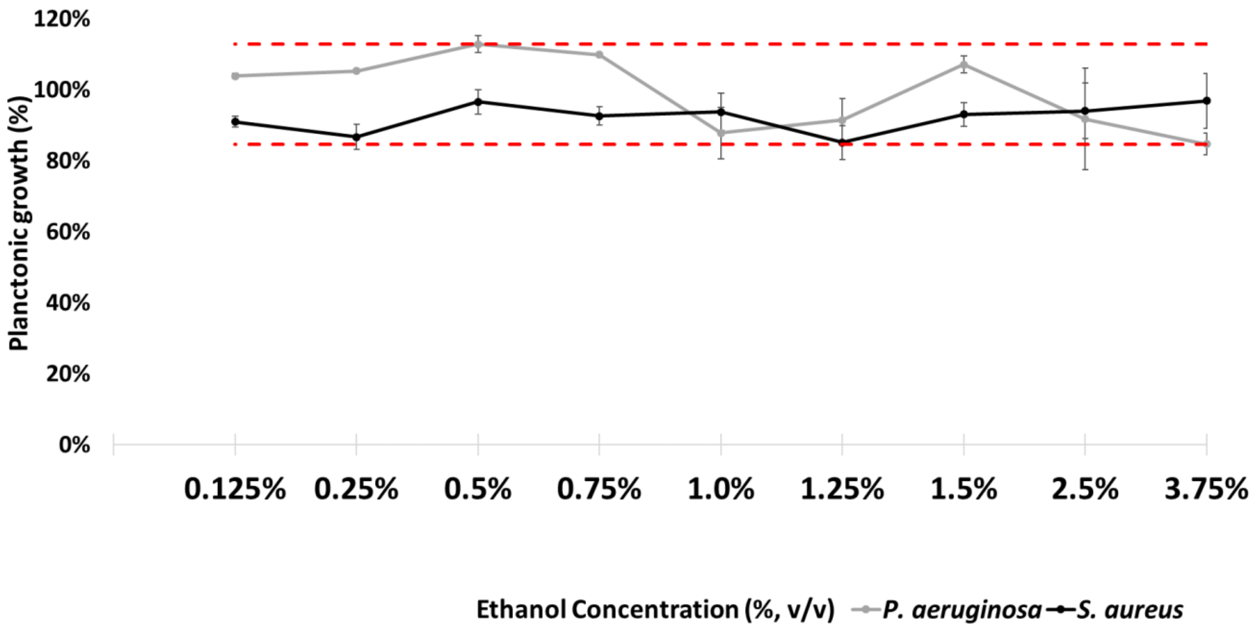

Supplementary Figure S1: Planktonic bacteria growth after ethanol exposure for 24 h. 100% corresponds to growth without ethanol. All data normalized to control without ethanol.

(A)

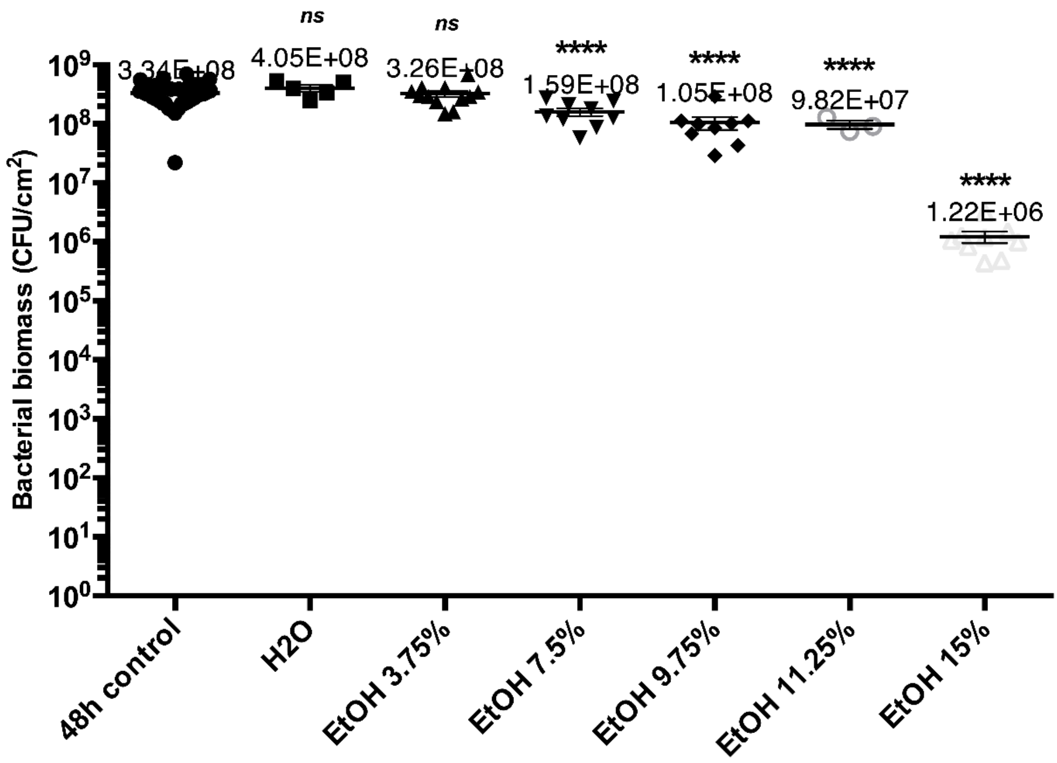

(B)

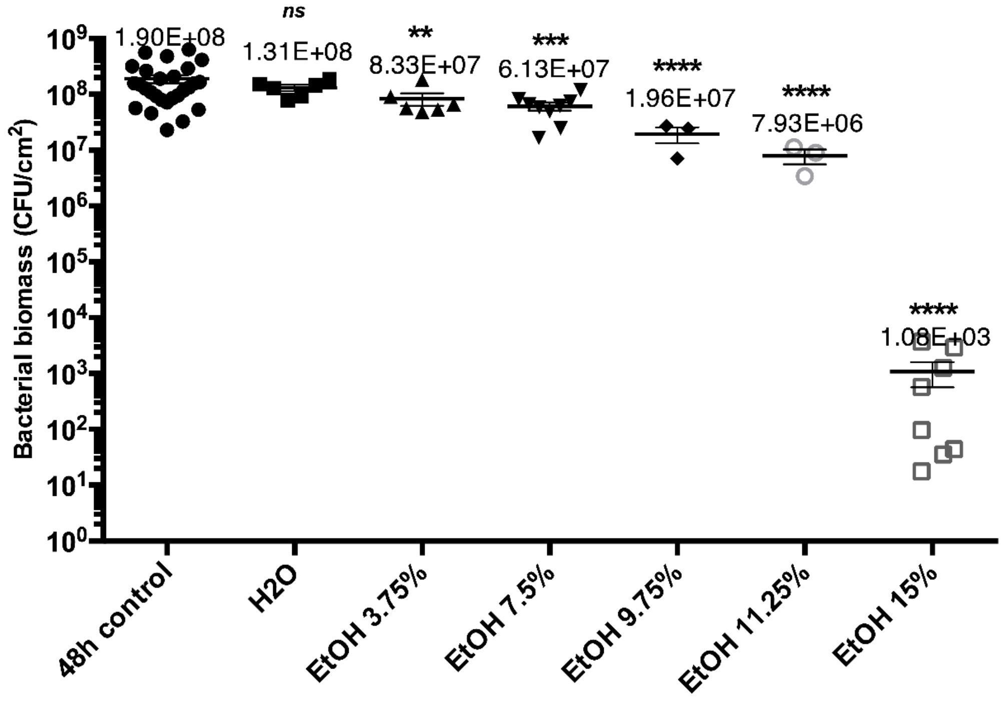

Supplementary Figure S2: Biofilm bacteria enumeration after ethanol exposure for 24 h. (**A**) *S. aureus* (**B**) *P. aeruginosa.*
